# Unraveling the Basis of Neonicotinoid Resistance in Whitefly Species Complex: Role of Endosymbiotic Bacteria and Insecticide Resistance Genes

**DOI:** 10.3389/fmicb.2022.901793

**Published:** 2022-06-23

**Authors:** Mritunjoy Barman, Snigdha Samanta, Gouranga Upadhyaya, Himanshu Thakur, Swati Chakraborty, Arunava Samanta, Jayanta Tarafdar

**Affiliations:** ^1^Department of Agricultural Entomology, Bidhan Chandra Krishi Viswavidyalaya, Mohanpur, India; ^2^Department of Biological Sciences, Indian Institute of Science Education and Research Kolkata, Kolkata, India; ^3^Department of Entomology, C.S.K. Himachal Pradesh Krishi Vishvavidyalaya, Palampur, India; ^4^Department of Plant Pathology, Bidhan Chandra Krishi Viswavidyalaya, Nadia, India

**Keywords:** whitefly, cryptic species, endosymbionts, genetic group, P450 monooxygenase, insecticide resistance, neonicotinoid, *mtCOI*

## Abstract

*Bemisia tabaci* (whitefly) is one of the most detrimental agricultural insect pests and vectors of many plant viruses distributed worldwide. Knowledge of the distribution patterns and insecticide resistance of this cryptic species is crucial for its management. In this study, genetic variation of mitochondrial cytochrome oxidase subunit 1 (*MtCoI*) gene of *B. tabaci* was analyzed followed by a study of the infection profile of various endosymbionts in 26 whitefly populations collected from West Bengal, India. Phylogenetic analysis revealed Asia I as the major cryptic species (65.38%), followed by Asia II 5, China 3, and Asia II 7, which were diversified into 20 different haplotypes. In addition to the primary endosymbiont (*C. poriera*), each of the four whitefly species showed a variable population of three secondary endosymbionts, majorly *Arsenophonus* with the highest infection rate (73.07%), followed by *Wolbachia* and *Rickettsia*. Further phylogenetic analyses revealed the presence of two subgroups of *Arsenophonus, viz*., A1 and A2, and one each in *Wolbachia* (W1) and *Rickettsia* (R3). Resistance to thiamethoxam, imidacloprid, and acetamiprid insecticides was analyzed for a clear picture of pesticide resistance status. The highest susceptibility was noted toward thiamethoxam (LC_50_ = 5.36 mg/L), followed by imidacloprid and acetamiprid. The whitefly population from Purulia and Hooghly districts bearing Asia II 7 and Asia II 5 cryptic species, respectively, shows maximum resistance. The differences in mean relative titer of four symbiotic bacteria among field populations varied considerably; however, a significant positive linear correlation was observed between the resistance level and relative titer of *Arsenophonus* and *Wolbachia* in the case of imidacloprid and thiamethoxam, while only *Wolbachia* was found in case of acetamiprid. Expression analysis demonstrated differential upregulation of insecticide resistance genes with Purulia and Hooghly populations showing maximally upregulated P450 genes. Moreover, thiamethoxam and imidacloprid resistance ratio (RR) showed a significant correlation with CYP6CM1, CYP6DZ7, and CYP4C64 genes, while acetamiprid RR correlated with CYP6CX1, CYP6DW2, CYP6DZ7, and CYP4C64 genes. Taken together, these findings suggested that P450 mono-oxygenase and symbiotic bacteria together affected whitefly resistance to neonicotinoids. Hence, a symbiont-oriented management programme could be a better alternative to control or delay resistance development in whitefly and can be used for pesticide clean-up in an agricultural field.

## Introduction

The whitefly, *Bemisia tabaci*, is an economically important agricultural pest causing huge damage to crops worldwide. They inflict damage to plants directly and as a vector of several hundred viruses, with a majority (>320 species) of them belonging to the genus Begomovirus and other economically important viruses belonging to the genera Ipomovirus, Carlavirus, Crinivirus, Torradovirus, and Polerovirus (Jones, 2003; Mugerwa et al., 2021). Whitefly is composed of a complex group of genetically distinct species and/or biotypes that differ substantively by host plant preference, virus transmitting ability, and insecticide resistance (De Barro et al., 2011; Barman et al., 2022). It is listed as one of the top 100 dreadful alien invasive species due to its virus transmission ability and wide host adaptability (Lowe et al., 2000). Dinsdale et al. (2010) proposed a 3.5% pair-wise genetic divergence of mitochondrial cytochrome oxidase I (*MtCoI*), which led to the identification of 44 distinct species of *B. tabaci*. Additionally, two new species, Asia II 13 and Spain 1, have also been recently reported (Kanakala and Ghanim, 2019). The Indian geographical regions unveil a huge diversity of *B. tabaci* with the presence of 10 cryptic species out of 46 recorded species so far (Rehman et al., 2021). Precise knowledge of the distribution pattern of this cryptic species is crucial for the efficient management of this notorious pest worldwide.

Nonetheless, increasing reports of resistance development in *B. tabaci* to various organophosphates, synthetic pyrethroids, and other neonicotinoid compounds have been surfacing (Peshin and Zhang, 2014; Naveen et al., 2017; Wang et al., 2020). Several countries, namely, China (Wang et al., 2010), India (Kranthi et al., 2002), Iran (Basij et al., 2017), Israel (Alon et al., 2008), Malaysia (Shadmany et al., 2015), Pakistan (Ahmad et al., 2010), and the USA (Prabhaker et al., 2014), have reported the development of insecticide resistance in whitefly to even compounds of novel chemistry. Intensive and frequent use of insecticides, selection pressure, and choice of poor insecticides may be some of the other factors rendering resistance in whitefly both at field and laboratory levels (Sethi and Dilawari, 2008; Pietri and Liang, 2018). The neonicotinoid group of compounds, represented by imidacloprid, thiamethoxam, and others, are majorly used insecticides in controlling the whitefly population worldwide, including in India (Sethi and Dilawari, 2008; Yang et al., 2013; Pang et al., 2020). However, several countries reported neonicotinoid resistance in whitefly due to the overuse of this chemical compound (Nauen et al., 2002; Ma et al., 2007; Luo et al., 2010; Naveen et al., 2017).

Researchers have observed that increased metabolic detoxification may be due to gene amplification, overexpression, and modification of gene coding proteins of major detoxifying enzymes, such as cytochrome P450 and glutathione S-transferases (GSTs) (Karunker et al., 2008; Feyereisen, 2011; Elzaki et al., 2017). The involvement of P450 genes in conferring resistance to several insecticides has been listed in insects, such as *Drosophila melanogaster* to DDT and imidacloprid, brown planthopper (BPH) to imidacloprid, and so on (Daborn et al., 2001; Garrood et al., 2016). Another most notable aspect of this complex is the bacterial symbionts that infect its members. Considerable evidence exists regarding microbe-mediated effects in whitefly, ranging from host survival, development, and nutritional fitness to even insecticide resistance (Su et al., 2014; Li et al., 2018). The *B. tabaci* species complex carries a primary endosymbiont *Candidatus Portiera aleyrodidarum* that occurs in all individuals and is located in specialized cells termed as bacteriocytes (Sloan and Moran, 2012). The secondary symbionts, namely, *Rickettsia* (Gottlieb et al., 2006), *Wolbachia* (Nirgianaki et al., 2003), *Hamiltonella, Arsenophonus* (Thao and Baumann, 2004; Sloan and Moran, 2012), *Cardinium* (Weeks et al., 2003), *Fritschea* (Everett et al., 2005), and *Hemipteriphilus* (Bing et al., 2013) have been reported to be present in *B. tabaci* populations around the world. Unlike primary endosymbionts which have a direct mutualistic relationship with the host, the secondary symbionts form a less stable partnership with their host. The infection pattern of these secondary symbionts is highly complex and varies according to the geographical regions (Zchori-Fein et al., 2014) and the different genetic groups (Chiel et al., 2007; Ghosh et al., 2015; Lestari et al., 2021).

The development of resistance is generally manifested by a failure to control the pests at the field level. A very few studies from India were involved in determining the field level resistance in *B. tabaci*; however, there was no in-depth study explaining the exact mechanism behind such resistance development. Hence, a precise understanding of the mechanism of insecticide resistance is of great significance for the management or delay in the development of resistance. Within this framework, this work hypothesized that there are differences in the resistance levels of whitefly populations in different regions toward insecticides like neonicotinoids, and this variation might be affected by the expression of genes associated with cytochrome P450 mono-oxygenase or variation of symbiont titres, either separately or together. To test this hypothesis, the following objectives were considered: (1) characterization and resistance level of the whitefly field population toward imidacloprid, thiamethoxam, and acetamiprid, (2) infection pattern and the relative titer of the endosymbiotic bacteria (*Portiera, Arsenophonus, Wolbachia*, and *Rickettsia*) associated with the whitefly field population, and (3) gene expression associated with cytochrome P450 mono-oxygenase (CYP6CM1, CYP6CX1, CYP6CX5, CYP6CX3, CYP6DZ4, CYP4C64, CYP6DZ7, and CYP6DW2) and their relationship with resistance level. Our finding, for the first time, shall shed light on field evolved resistance in different whitefly cryptic species toward popularly used neonicotinoids, further attempting to predict the mechanism behind such resistance development, in particular, the involvement of endosymbionts and P450 genes.

## Methods

### Whitefly Collection

The samples of *B. tabaci* used in the current study were collected from different locations across Bengal province, India (as detailed in Supplementary Table S1). Adult whiteflies (mixed sex) were collected from various crop plants (chili, eggplant, cucumber, tomato, lady's finger, and pointed gourd) and weed plants (cida and wild brinjal). The flies were pooled together inside ventilated insect-proof cages on the basis of location and further reared in the Molecular Biology laboratory, Directorate of Research, BCKV.

### Detection of Whitefly Genetic Groups and Endosymbionts

The genetic group identity of *B. tabaci* field population was examined by random sampling of 10 adult flies for each population using PCR amplification of the *MtCoI* gene and sequencing as described by Dinsdale et al. (2010). Genomic DNA was extracted from each specimen as per the manufacturer's instructions (Invitrogen, USA). PCR amplification using 20 ng of insect DNA was carried out in Veriti 96-Well Thermal Cycler (Applied Biosystems, USA). The endosymbionts (*Portiera, Wolbachia, Arsenophonus*, and *Rickettsia*) in the collected whitefly populations were also determined using specific primers (as listed in Supplementary Table S2). The amplified PCR products were excised from the gel and purified using the XcelGen DNA Gel/ PCR Purification mini kit (Xcelris genomics, India) following the manufacturer's instructions. The DNA sequence was obtained through Sanger dideoxy sequencing method from Chromus Biotech Laboratory, India.

### Genetic Structuring and Phylogenetic Analysis

Different neutrality statistics were performed to examine the demographic history of whitefly and their endosymbionts. Tajima's *D* (Tajima, 1989) and Fu's Fs (Fu, 1997) statistics were subsequently calculated to check whether the *COI* conformed to the expectations of neutrality by DnaSP (Librado and Rozas, 2009). The population pair-wise *F*-statistics (*F*_ST_) and analysis of molecular variance (AMOVA) were calculated with the help of Arlequin v.3.5 (Excofer and Lischer, 2010). The haplotype network of the sequences was examined using a minimum spanning network relationship with the help of popART software. Multiple alignments of all the nucleotide sequences of whiteflies and endosymbionts were conducted using ClustalW, and analysis was done in Mega-X (Kimura, 1980; Kumar et al., 2018). The tree was verified using the maximum likelihood (ML) model, and a total 1,000 of bootstrap replicates were performed. The tree was processed through iTol (version 6.4.3) for graphical representation. All the nucleotide sequences of the whitefly population and endosymbionts were submitted to the NCBI GenBank database.

### Bioassay of Insecticides

To determine the current susceptibility status and resistant gene expression of whitefly, the field populations from the five most important regions (one population each from Kalimpong, Midnapore, Hooghly, Malda, and Purulia) were selected (as listed in Supplementary Table S3). For the bioassay, a total of approximately 800 adult flies were collected per location, and around 650 of them were used immediately in the bioassay study. The whitefly population collected from the university farm (B.C.K.V., Kalyani, India) was chosen to be reared for 10 additional generations without any exposure to insecticides in the laboratory and was considered as the susceptible population (SP). Three insecticides, namely, imidacloprid 17.8 SL (Confidor) obtained from Bayer Co., Ltd., thiamethoxam 25 WG (Actara) from Syngenta Chemical Ltd., and acetamiprid 20SP (Pyramid) from Hifield-AG Chem. Pvt. Ltd., were used for bioassay studies against the adult whitefly population. The toxicity of imidacloprid, thiamethoxam, and acetamiprid was evaluated following a modified leaf dip bioassay method by the Insecticide Resistance Action Committee (Naveen et al., 2017). To evaluate the toxicity, final doses were decided based on the prior experiments conducted on lab populations, and five different concentrations of each insecticide were used. In each insecticide treatment, fresh brinjal leaves (5 × 5 cm^2^) were dipped in the respective insecticide dilutions for 30 ± 2 s in a corning glass Petri plate (dia. = 15 cm) and air-dried, whereas the control leaves were dipped in water alone. After drying the solution on the leaf surface, the treated leaves were transferred to the corning glass Petri plate (dia. = 9 cm) containing a thin 2% agar layer at the bottom. Each treatment was replicated five times, and six adult whiteflies were released per replication (thirty in each treatment) and plates were covered with ventilated lids. The experiment was conducted under laboratory conditions (26 ± 1°C, 70–80% RH, 16L: 8D photoperiod), and the observations regarding the mortality were taken at 72 h after feeding (HAF).

### Symbiont Quantification and Expression Analyses of Insecticide Resistance Genes

Total genomic DNA (for quantification of symbiont titer) and RNA (for gene expression) were extracted from field-collected whitefly (adult) population using Insect DNA and RNA Isolation Kit (Thermo Fisher Scientific, USA) following the manufacturer's protocol. Quantity was assessed with a Qubit Flex Fluorometer, and cDNA was prepared using 1 μl of total RNA according to the manufacturer's instructions (GeneSure H-Minus First Strand cDNA Synthesis Kit, Genetix Biotech Asia Pvt. Ltd.).

The relative abundance of targeted symbionts (*Portiera, Arsenophonus, Wolbachia*, and *Rickettsia*) and expression pattern of cytochrome P450 genes, namely, CYP6CM1, CYP6CX1, CYP6CX5, CYP6CX3, CYP6DZ4, CYP4C64, CYP6DZ7, and CYP6DW2, were examined using qRT-PCR (Wang et al., 2020). qRT-PCR was performed in Agilent Technologies Stratagene (Model-Mx3000P) using SYBR Green Master Mix (Applied Biosystems, USA). Primer details and annealing temperatures are mentioned in Supplementary Table S2. The relative expression of each target gene was calculated by the 2^−Δ*ΔCt*^ method (Livak and Schmittgen, 2001). The Actin gene was used as an internal control.

### Statistical Analysis

To determine the lethal concentration (LC) values, the observations recorded on mortality data were corrected using Abbott's formula (Abbott, 1925). The data were subjected to probit analysis (Finney, 1971) using SPSS. The resistance ratio (RR) for each population was calculated by the ratio: LC_50_ value of the population/LC_50_ value of the susceptible population. The RRs were then classified as follows: RR < 5 indicated low resistance; RR = 5–10 indicated moderate resistance, and RR > 10 indicated high resistance (Mazzarri and Georghiou, 1995; World Health Organization, 2016). The qRT-PCR experiments were conducted at least thrice with a minimum of three individual biological replicates. Data have been represented as mean ± SD (*n* = 3) values. Statistical significance was analyzed using the GraphPad Prism 8.0 software by one-way analysis of variance (ANOVA), followed by Dunnett's multiple comparison test. Significant differences with the susceptible population have been represented as ^*^p < 0.01, ^**^p < 0.001, and ^***^p < 0.0001. Linear Model II regression analysis was used to determine the functional relationship between the resistance level of the population and the mean normalized expression value of each gene and endosymbiont in different populations using SPSS (SPSS for Windows, Rel. 17.0.0 2009. Chicago: SPSS Inc.).

## Results

### Exploring the Prevalence of Different Genetic Groups of Whitefly and Their Symbionts

**Figure 1:**
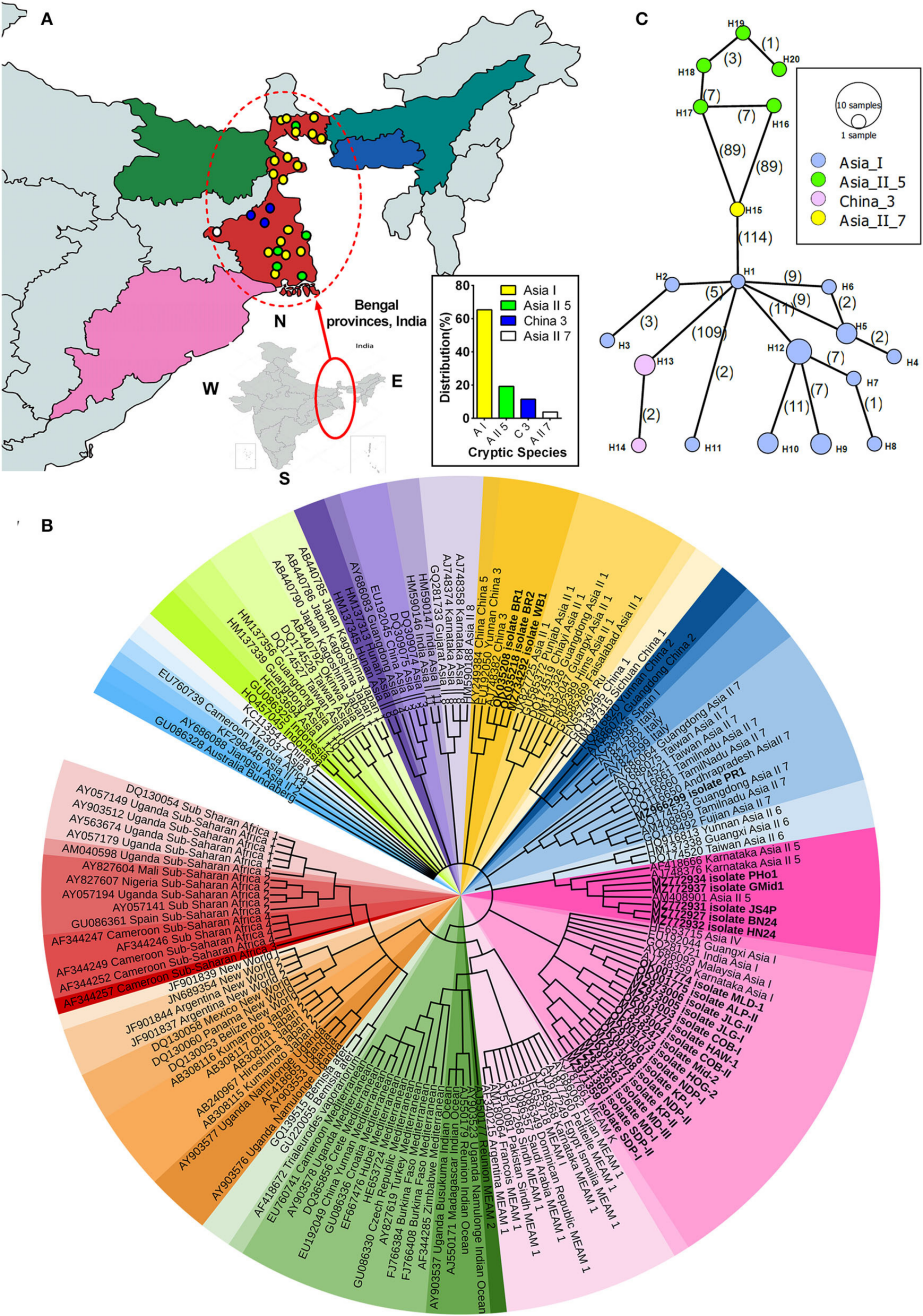
Phylogenetic analysis of whitefly (*Bemisia tabaci*) based on *COI* gene. **(A)** Map indicating different locations of *B. tabaci* sampling and distribution across different genetic groups in Bengal provinces, India. Inset shows the percentage distribution of various cryptic species of *B. tabaci* from collected specimens. **(B)** The maximum likelihood phylogenetic tree of the *COI* sequences of *Bemisia tabaci* collected from different locations. The bold text *COI* sequences represent collected samples used in this study, and the rest represent reference sequences obtained from the GenBank database. A total of 159 nucleotide sequences were selected to construct the tree, wherein *Bemisia afer* and *Trialeurodes vaporariorom* were taken as an out-group. Different color sheds indicate the different cryptic species of whitefly distributed worldwide. Hasegawa-Kishnio-Yano HKY850 model and gamma distribution rate of variation among sites were implemented to construct the phylogenetic tree in MEGA X. **(C)** Minimum spanning network of different *B. tabaci* cryptic species haplotypes from the studied samples. The four different colors indicate the haplotype of *B. tabaci* cryptic species. Each circle represents a unique haplotype, the frequency of which is proportional to the diameter of the circle. The number of mutations is represented by numbers in parenthesis.

In totality, 26 whitefly populations collected from different host crops across different regions of Bengal province (as shown in Figure 1A) were identified using the primer pair (C1-J-2195 F/L2-N-3014 R) of the universal *Mt-co1* gene. The phylogenetic analysis of the determined *COI* sequences divided *B. tabaci* into four different cryptic species, with Asia I being the most predominant species followed by Asia II 5, China 3, and Asia II 7 at rates of 65.38, 19.23, 11.53, and 3.84%, respectively (Figure 1A inset). To show the distribution of collected samples, a maximum likelihood phylogenetic tree of the *COI* sequences of *Bemisia tabaci* collected from different locations has been drawn with previously reported GenBank sequences, wherein the bold text *COI* sequences represent collected samples used in this study (Figure 1B). Subsequently, diagnostic PCR confirmed the presence of primary endosymbiont *Portiera* and secondary endosymbionts *Wolbachia, Arsenophonus*, and *Rickettsia* in the selected whitefly population.

### Study of Evolutionary Relationship Advocates Evident Genetic Diversity and Expansion Nature of Whitefly Cryptic Species

The pair-wise divergence in the *MtCoI* nucleotide sequence among the four identified genetic groups varied both at intraspecific and interspecific levels (Supplementary Table S4). Intraspecific variation was recorded to be the highest in Asia I (0.24–2.66%), and the lowest was observed in China 3 (0.11–0.23%). While considering the interspecific variation, Asia I and Asia II 5 (17.47–20.80%) were higher than that of Asia I and China 3 (14.63–17.09%). However, the lowest variation was observed between China 3 and Asia II 7 (16.95–17.30%).

Among the 20 haplotypes (based on *MtCoI* sequence), the highest number was recorded in Asia I (12), followed by Asia II 5 (5) and China 3 (2) (Supplementary Table S5). Asia II 7 was excluded from the genetic diversity study, as only one such sample was found in our survey. Haplotype diversity was higher in Asia II 5 (1) when compared to Asia I (0.956), whereas nucleotide diversity was higher in Asia I (0.014) in comparison to Asia II 5 (0.008). Results obtained from the neutrality test (Fu and Li's D^*^, Fu and Li's F^*^, and Tajima's *D*) recorded a significant negative value for Asia II 5 and China 3, indicating that these two cryptic species across the regions might be undergoing expansion.

Minimum spanning network analysis indicated four cryptic species to be diversified into 20 haplotypes, which are quite distant from one another (Figure 1C). Among the 12 haplotypes of Asia I, H1 occupied the central position of the network and was the linking haplotype between China 3 (H13) and Asia II 7 (H15) with the mutational differences of 109 and 114, respectively. Furthermore, hierarchical AMOVA revealed that most of the evident genetic diversity is collectively accredited to highly significant genetic differences between the four whitefly cryptic species (Fst: 91.83%; *P* < 0.001), and the haplotypes within these cryptic species contributed to 8.16% of the variation (Supplementary Table S6). Moreover, the genetic differentiation among the population (pair-wise Fst value) was >0.90 for all the four cryptic species (Supplementary Table S7).

### Variation in the Infection Pattern of Endosymbiotic Bacteria Is Governed by *B. tabaci* Genetic Group

Primary endosymbiont (*C. poriera*) was present in all the test samples; however, the infection profiles of three secondary endosymbionts (*Arsenophonus, Wolbachia*, and *Rickettsia*) showed a substantial variation in each of the four identified cryptic whitefly species. Overall, *Arsenophonus* showed the highest infection rate (73.07%), followed by *Rickettsia* (65.38%) and *Wolbachia* (53.84%). *Arsenophonus* infection was found in Asia I, Asia II 5, and Asia II 7 population but not in China 3 population, with Asia I bearing the highest infection rate (Supplementary Table S8).

**Figure 2:**
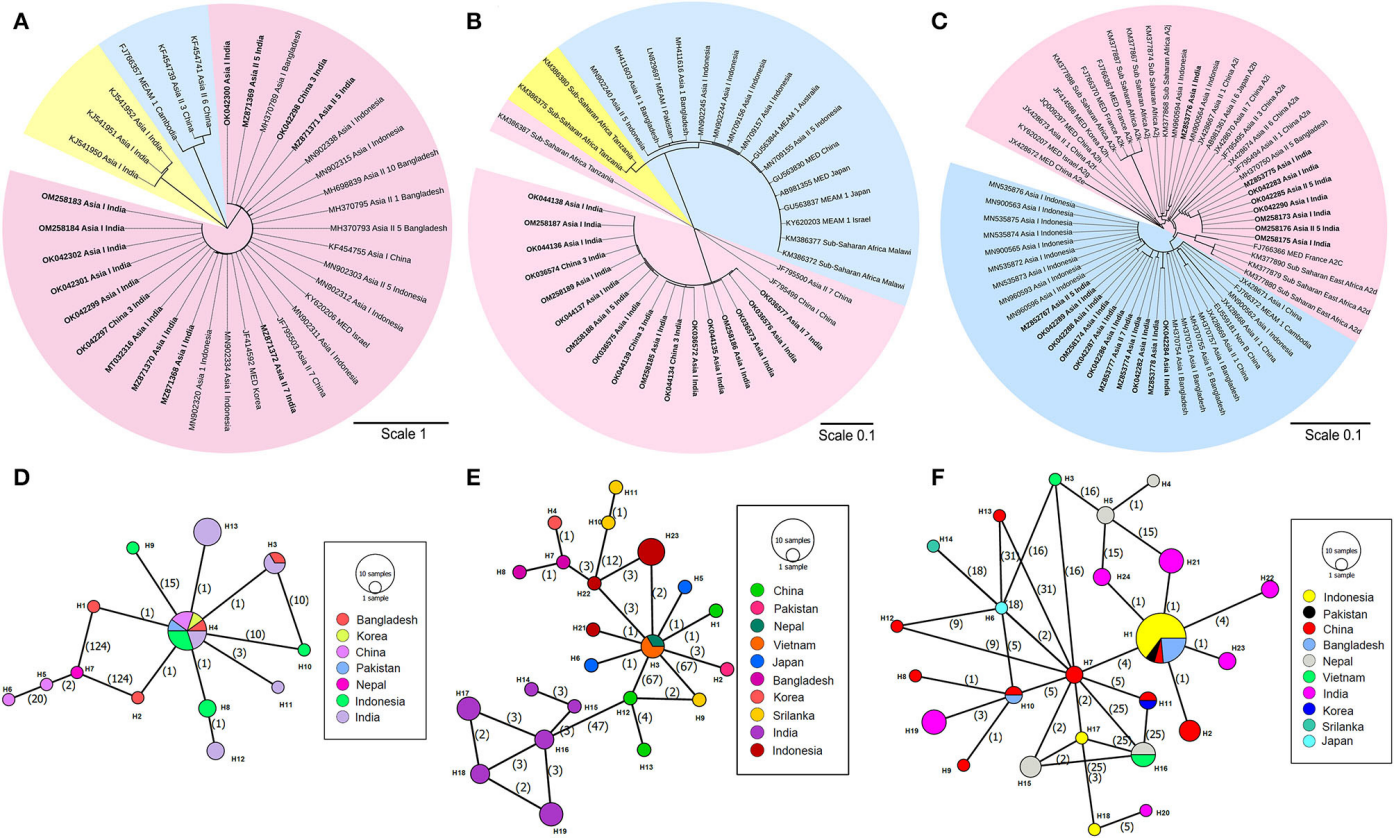
Phylogenetic tree and network analysis of secondary endosymbiont based on 16S rDNA (*Wolbachia* and *Rickettsia*) and 23S rDNA (*Arsenophonus*) gene segments. **(A)** The maximum likelihood phylogenetic tree of the 16S rDNA sequences of *Wolbachia* sp. infecting different whitefly populations. Different color shades indicate the subgroup of *Wolbachia* species (W1 subgroup-pink shade, W2 subgroup-blue shade, W3 subgroup-yellow shade) distributed worldwide. **(B)** The maximum likelihood phylogenetic tree of the 16S rDNA sequences of *Rickettsia* sp. infecting different whitefly populations. Different color shades indicate the subgroup of *Rickettsia* species (R1 subgroup-blue shade, R2 subgroup-yellow shade, and R3 subgroup-pink shade) distributed worldwide. **(C)** The maximum likelihood phylogenetic tree of the 23S rDNA sequences of *Arsenophonus* sp. infecting different whitefly populations. Different color shades indicate the subgroup of *Arsenophonus* species (A1 subgroup-blue shade and A2 subgroup-pink shade) distributed worldwide. Hasegawa-Kishnio-Yano HKY850 model and gamma distribution rate of variation among sites were implemented to construct all the phylogenetic trees in MEGA X. Minimum spanning network of different *Wolbachia* species **(D)**, *Rickettsia* species **(E)**, and *Arsenophonus* species **(F)** present in collected whitefly samples from India and other Asian countries. The different colors indicate the haplotype of each species. Each circle represents a unique haplotype, the frequency of which is proportional to the diameter of the circle. The number of mutations is represented by numbers in parenthesis.

Furthermore, based on the criterion of Kanakala and Ghanim (2019), the secondary endosymbionts were classified at the subgroup level. The phylogenetic tree analysis revealed the presence of only one subgroup of *Wolbachia* (W1) and one subgroup of *Rickettsia* (R3) that infected all the four cryptic whitefly species (Figures 2A,B, respectively**)**. The phylogenetic tree also revealed the presence of two subgroups of *Arsenophonus*: A1 and A2 (Figure 2C). The infection rate of A1 was always higher than A2 in Asia I population, which was in contrast to Asia II 5 population, where A2 was higher. The infection rate of *Wolbachia* was 40–100%, with the highest rate occurring in Asia II 7 and China 3 populations. The infection rate of *Rickettsia* was the highest in China 3 and Asia II 7 populations, followed by Asia I and Asia II 5 populations, respectively. Thus, as observed, all the whitefly individuals were infected with the variable combinations of the three secondary endosymbionts (Supplementary Table S9).

### Comparative Network Analysis Revealed a Close Relationship Between the Symbiont Haplotypes

In the current study, an intriguing facet was to comprehend the genetic variation of secondary symbionts with that of the symbionts in other whitefly species complex. This is anticipated to further assist in the discovery of the population demography of whitefly and the relationship between different sub-populations. The haplotype diversity and nucleotide diversity of *Wolbachia* in our collected samples were 0.803 and 0.006, respectively (Supplementary Table S10), which further diversified into five different haplotypes (Figure 2D). On the other hand, the neutrality test revealed the haplotype and nucleotide diversity of *Rickettsia* to be 0.879 and 0.003, respectively, with the presence of six haplotypes (Figure 2E). Subsequently, the presence of six haplotypes was observed among the *Arsenophonus* sequences: the A2 subgroup of *Arsenophonus* had only two haplotypes, and the A1 subgroup consisted of four haplotypes (Figure 2F). Interestingly, the network analysis also revealed a considerable overlap among the symbiont haplotypes from different Asian countries, sometimes with a little variation.

The demographic history when analyzed using Fu and Li's D^*^, Fu and Li's F^*^, and Tajima's *D* tests, each returned a positive value for the sequences of *Arsenophonus* and *Rickettsia* (interpreted as a sudden population contraction) used in the current study, but since the value was not significant, the null hypothesis regarding the neutrality of the population is accepted. However, Tajima's *D* test statistic showed a negative but non-significant value for *Wolbachia* specimens. Overall, the Tajima's *D* values were negative but not significant in all the symbionts outside India. As a test for a recent population expansion, the mismatch distribution analysis indicated a non-significant multimodal distribution for the genes of three endosymbiotic bacteria. However, the distribution observed was higher than the expected one.

### Bioassay Studies Indicate Variation in Toxicity of Selected Neonicotinoids Toward *B. tabaci* Field Population

**Figure 3:**
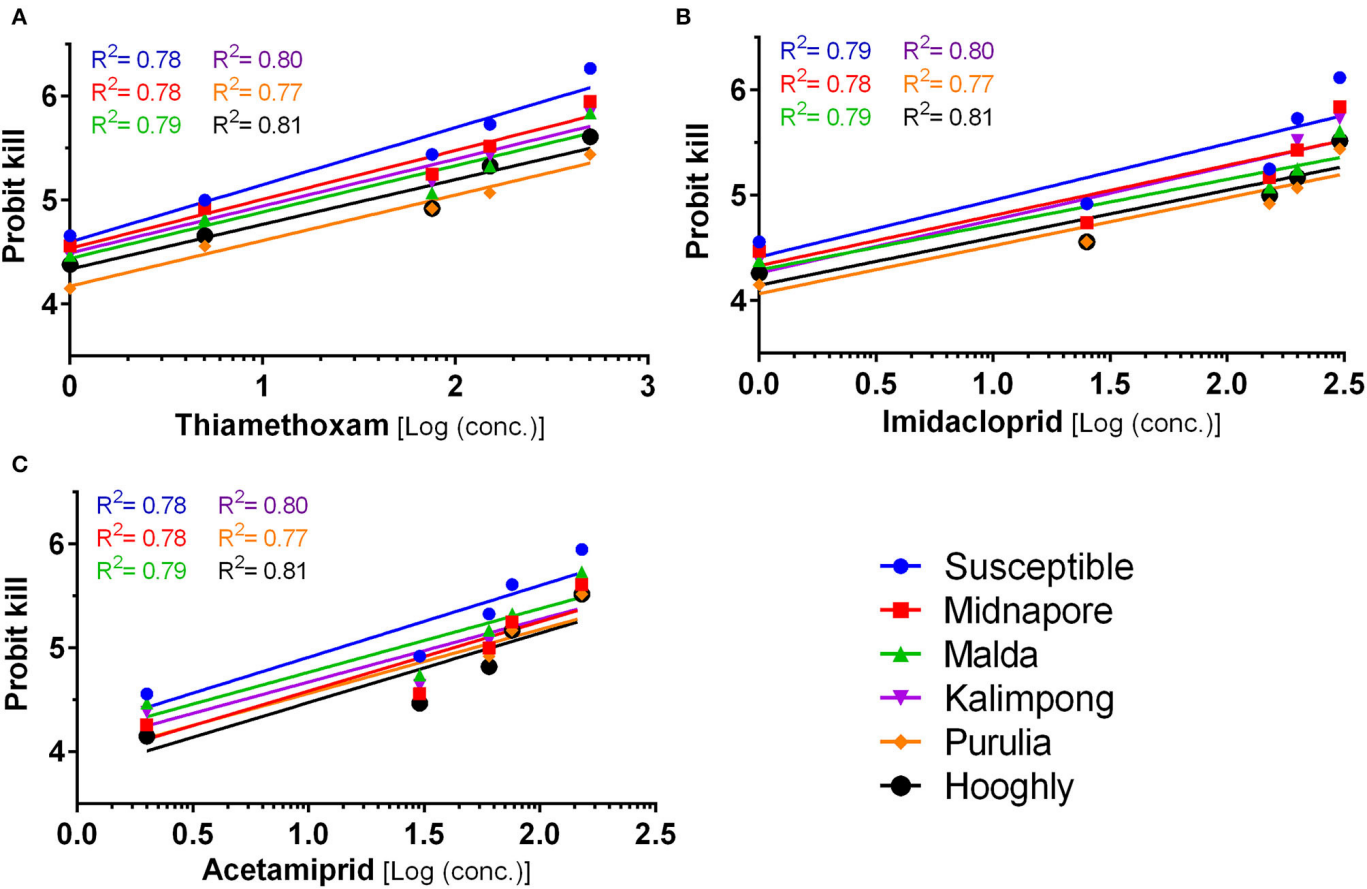
Mortality response of whitefly (*Bemisia tabaci*) population to insecticides. Increasing concentrations of three insecticides, thiamethoxam **(A)**, imidacloprid **(B)**, and acetamiprid **(C)** was tested against different populations of whitefly labeled with separately colored lines, as marked in the legend to analyse the mortality response. The *X*-axis represents the logarithmic dose of every insecticide concentration tested, while the *Y*-axis represents the probit kill values. The straight line represents the best fitting probit regression line between insecticide log dose and mortality probits (working probits) for adult whitefly. The *R*^2^ of each individual line in the graphs represents the regression coefficient. Slopes of the lines are given in Supplementary Table S11.

The susceptibility of six whitefly populations has been evaluated against three different insecticides, namely, thiamethoxam, imidacloprid, and acetamiprid (Figure 3). The results (detailed in Supplementary Table S11) indicate that the toxicity (LC_50_) level varies between 5.36 and 52.14 mg/L for thiamethoxam, 12.87 and 112.09 mg/L for imidacloprid, and 13.49 and 60.04 mg/L for acetamiprid, signifying the varying susceptibility of these populations toward the neonicotinoid group of insecticides. Based on the LC_50_ value of the susceptible laboratory population, thiamethoxam (LC_50_ = 5.36 mg/L) was found to be the most toxic, followed by imidacloprid (12.87 mg/L) and acetamiprid (13.49 mg/L). The populations belonging to Midnapore and Kalimpong exhibited a similar toxicity trend against these insecticides **(**detailed in Supplementary Table S11). On the contrary, populations from Hooghly, Malda, and Purulia followed a slightly different trend, wherein the highest toxicity was observed in the case of thiamethoxam, followed by acetamiprid (LC_50_ = 60.04, 24.37, and 52.23 mg/L) and imidacloprid (LC_50_ = 77.79, 44.53, and 112.09 mg/L, respectively) (detailed in Supplementary Table S11).

On comparing the susceptibility of different populations against an insecticide, the data indicated that the resistance ratio (RR value) is in the range of 1.00–9.73, 1.00–8.71, and 1.00–4.45 for thiamethoxam, imidacloprid, and acetamiprid, respectively (Supplementary Table S11). The maximum RR value for thiamethoxam was recorded in the population of Purulia (9.73), where the lowest (1 ppm) and highest (300 ppm) tested concentration resulted in the working probit kill of 4.15 and 5.44, respectively. On the other hand, susceptible (Nadia population) working probit kill was 4.66 and 6.27, respectively, for the same concentrations (Figure 3A). Toxicity comparison with the laboratory population for imidacloprid indicated the highest RR value (8.71) for the Purulia population (Supplementary Table S11). The population of Purulia showed working probit kill values of 4.15 and 5.44 for the lowest (2 ppm) and highest (450 ppm) test concentrations of imidacloprid, respectively, whereas the susceptible population (Nadia) showed working probit kill values of 4.56 and 6.12, respectively (Figure 3B). In the case of acetamiprid, the maximum RR value (4.45) was observed in the Hooghly population (Supplementary Table S11), wherein the lowest (4 ppm) and highest (400 ppm) tested concentrations resulted in a working probit kill of 4.15 and 5.52 (Figure 3C), respectively. Based on the guidelines by World Health Organization (2016) and studies by Mazzarri and Georghiou, 1995 and Yi et al., 2020, the resistance to thiamethoxam and imidacloprid can be considered to exist at a moderate level (i.e., 8.7–9.7), while resistance to acetamiprid was comparatively lower (i.e., 4.45).

Therefore, the bioassay study indicates that the whitefly population from Purulia and Hooghly, consisting of Asia II 7 and Asia II 5 cryptic species populations, respectively, have developed maximum resistance toward the selected neonicotinoids (Supplementary Table S3).

### Relative Titer of *Arsenophonus* and *Wolbachia* Correlated With Resistance Ratio of Three Neonicotinoid Insecticides

**Figure 4:**
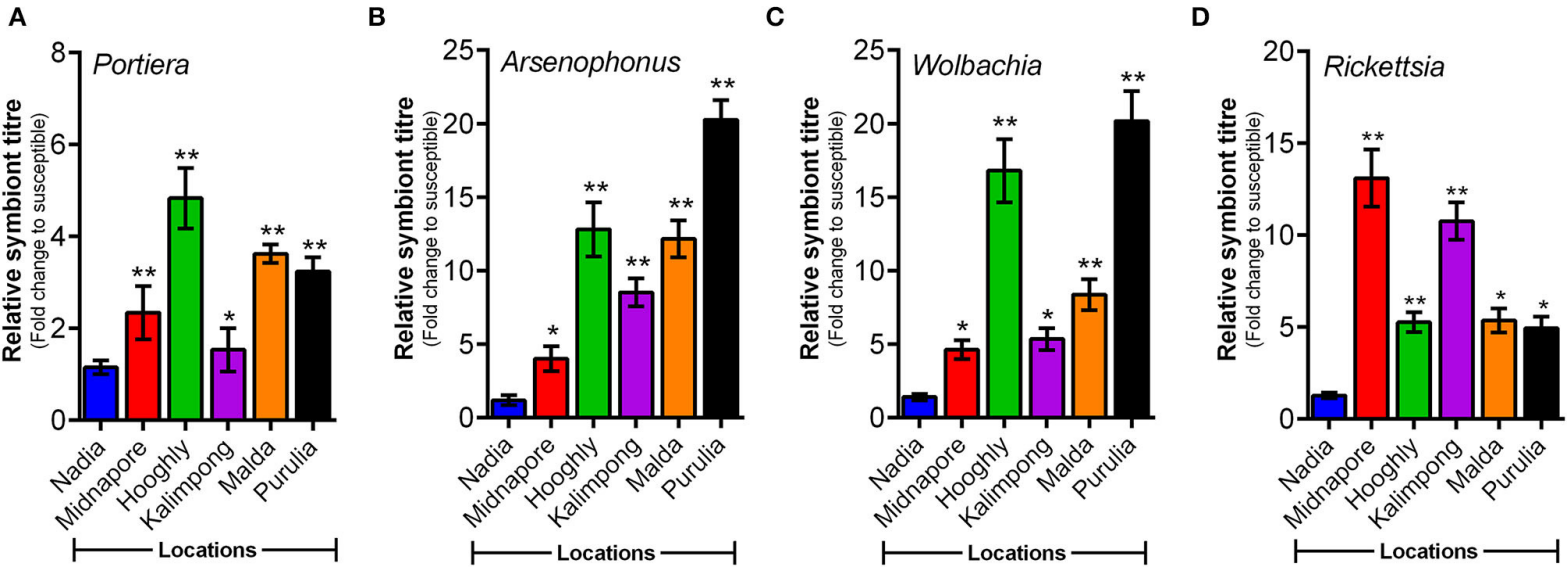
Relative titer of endosymbiotic bacteria (normalized to the whitefly's nuclear β-actin gene) in different whitefly populations as quantified by qPCR. Titer of four endosymbiotic bacteria (*Portiera, Arsenophonus, Wolbachia*, and *Rickettsia*) in whitefly populations (as listed along the X-axes). **(A)** *Portiera*, **(B)** *Arsenophonus*, **(C)** *Wolbachia*, and **(D)** *Rickettsia*. Values represent mean ± SE values for three independent replicates. Statistical significance of the experimental sets as compared to the susceptible population has been marked with ***p ≤ 0.0001, **p ≤ 0.001, and *p ≤ 0.01.

A comparative study involving symbiont titres in different field populations of whitefly revealed varying patterns in their quantitative analysis. The relative titer of P-symbiont (*Portiera*) differed significantly among the experimental whitefly population. Significantly higher members of *Portiera* were observed in the population of Hooghly, followed by the population of Malda and Purulia when compared to the susceptible reference population (Nadia) (Figure 4A). The results also revealed varying patterns in titer level of S-endosymbionts, that is, *Arsenophonus, Wolbachia*, and *Rickettsia*. A significantly higher abundance of *Arsenophonus* and *Wolbachia* was observed in the population of Purulia, followed by the population of Hooghly and Malda when compared to the reference population (Figures 4B,C). Contrary to the other symbionts, the maximum titer of *Rickettsia* was observed in the population of Midnapore and Kalimpong (Figure 4D).

**Figure 5:**
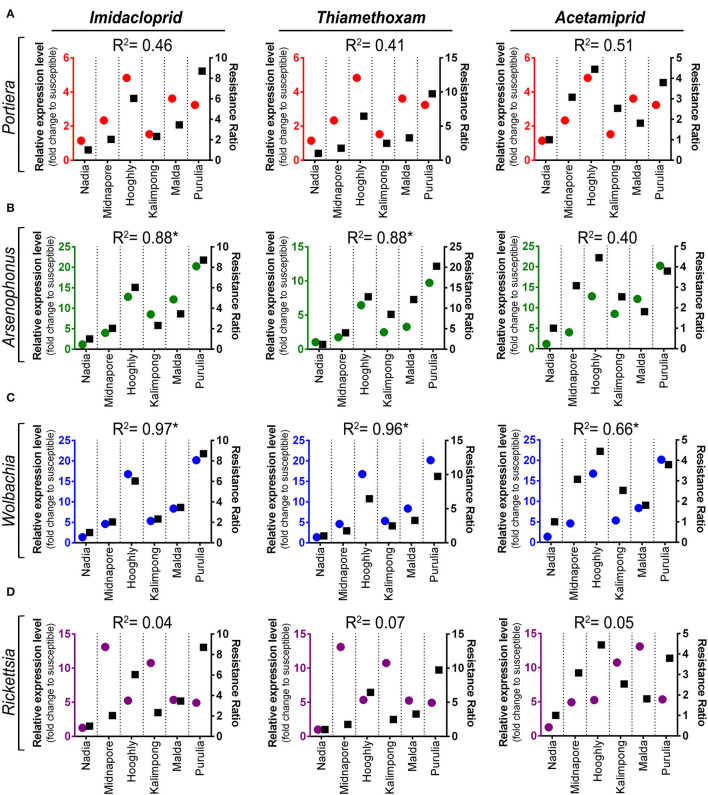
Linear regression analysis between insecticides resistance level and relative symbiont titer of different whitefly populations. Linear regression analysis was used to test for a functional relationship between the resistance level of each insecticide (imidacloprid, thiamethoxam, and acetamiprid) and the mean normalized expression value of each symbiotic bacteria in different whitefly populations: **(A)** *Portiera*, **(B)** *Arsenophonus*, **(C)** *Wolbachia*, and **(D)** *Rickettsia*. Circles in each graph represent the relative titer of each symbiont (marked on the left *Y*-axis), while squares depict the resistance ratio of each insecticide (labeled on the right *Y*-axis).

The correlation coefficient between resistance ratio (RR) values of whitefly field population and relative abundance of symbiont titer is presented in Figures 5A–D. The titer level of *Arsenophonus* was significantly correlated with RR of imidacloprid and thiamethoxam, with correlation coefficients of 0.88 (*p* = 0.005) and 0.88 (*p* = 0.006), respectively (Figure 5B). Moreover, the resistant ratio of all the three neonicotinoids exhibited a positive correlation with the titer level of *Wolbachia*. The correlation coefficients were 0.97 (*p* = < 0.001) for imidacloprid, 0.96 (*p* = <0.001) for thiamethoxam, and 0.65 (*p* = 0.050) for acetamiprid, respectively (Figure 5C). The titer level of *Portiera* shows positive but no significant relation with RR among all the treated insecticides.

### Upregulation of CYP6DZ7 and CYP4C64 Genes Correlated Well With Resistance Ratio of Neonicotinoid Insecticides

**Figure 6:**
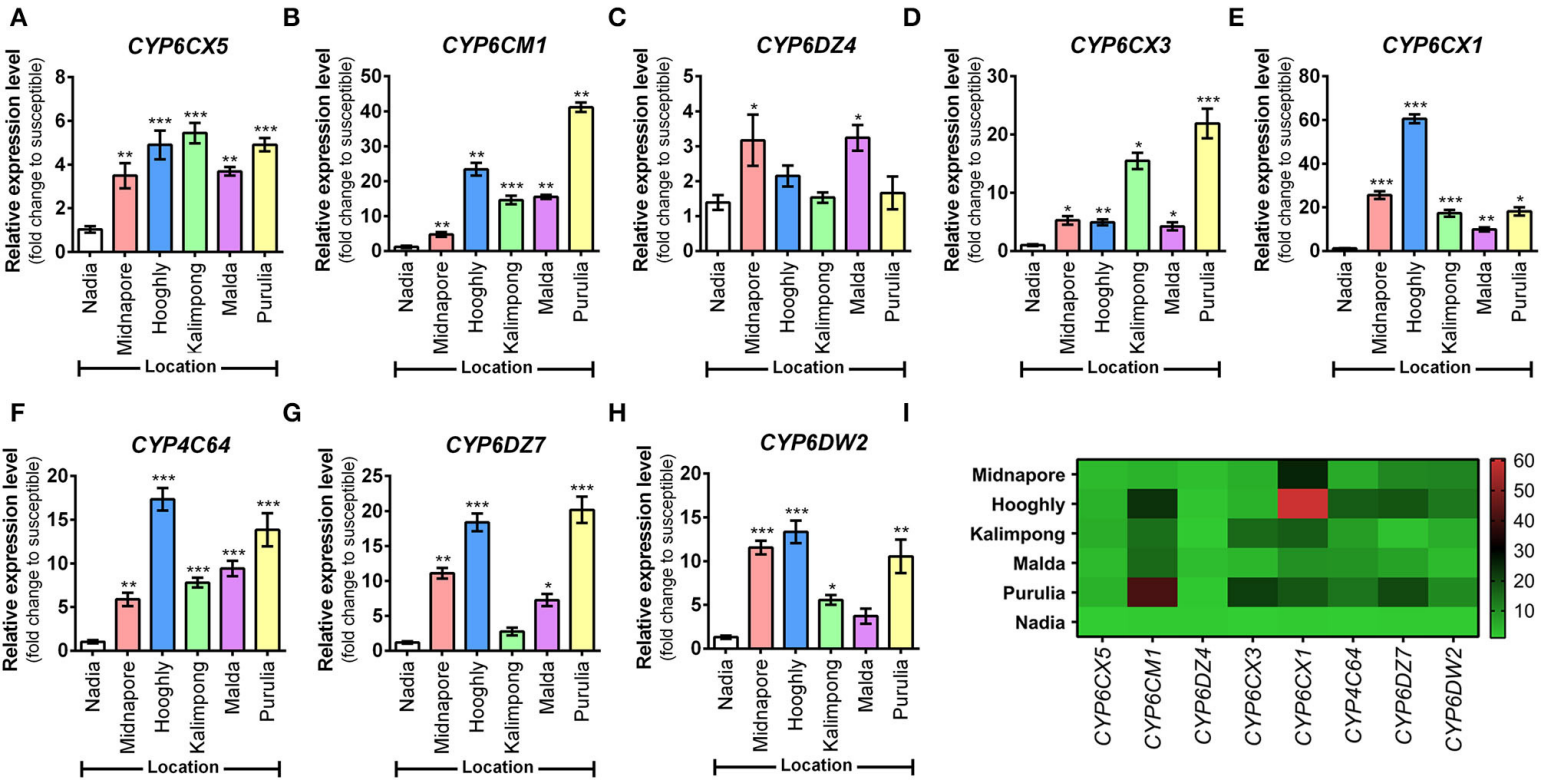
Expression analysis of different cytochrome P450 genes in whitefly (*Bemisia tabaci*) population. Expression profiles of eight P450 genes governing insecticide resistance in whitefly populations (as listed along the *X*-axes). **(A)** CYP6CX5, **(B)** CYP6CM1, **(C)** CYP6DZ4, **(D)** CYP6CX3, **(E)** CYP6CX1, **(F)** CYP4C64, **(G)** CYP6DZ7, and **(H)** CYP6DW2. Relative gene expression was measured by qRT-PCR analysis. Nuclear β-actin has been used as the internal control. The Ct values for tested genes were normalized to the Ct value of actin and calculated relative to a calibrator using the formula 2^−Δ*ΔCt*^. Values represent mean ± SE for three independent replicates. Statistical significance of the experimental sets as compared to the susceptible population has been marked with ***p ≤ 0.0001, **p ≤ 0.001, and *p < 0.01. **(I)** Heatmap represents an overview of the expression level of each gene in a variable whitefly population, with the color gradient provided alongside. Gene expression profiles are represented as a relative expression when compared to the susceptible population, obtained from the qRT-PCR experiment.

The expression of eight cytochrome P450 genes was compared among five whitefly populations against the susceptible reference population (Nadia), wherein upregulation of all the eight genes was noted at varying degrees. In the case of CYP6CX5, Kalimpong, Purulia, and Hooghly populations showed maximum upregulation (4–5-fold), followed by Malda and Midnapore populations that depicted a 3-fold upregulation (Figure 6A). Subsequently, for the CYP6CM1 transcript level, the Purulia population exhibited the highest upregulation (41-fold), followed by Hooghly (23-fold) and Malda (15-fold) (Figure 6B). The relative expression of CYP6DZ4 was low in all the populations, with maximum expression observed in the Malda population (3-fold) (Figure 6C). Next, for the CYP6CX3 transcript level, the Purulia population exhibited the highest upregulation (21-fold), followed by Kalimpong (15-fold) and Midnapore (5-fold) (Figure 6D). Both CYP6CX1 and CYP4C64 transcript levels were maximum for the Hooghly population, with an increase of 60-fold and 17-fold, respectively (Figures 6E,F). Lastly, the relative expression of CYP6DZ7 and CYP6DW2 was moderately high in the population of Purulia and Hooghly (20-fold and 18-fold; 10-fold and 13-fold, respectively) (Figures 6G,H). Overall, the results show that Purulia and Hooghly populations harbored maximally upregulated insecticide resistance P450 genes.

**Figure 7:**
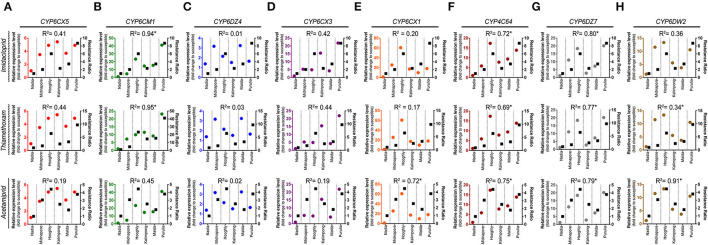
Linear regression analysis between insecticide resistance level and relative expression of insecticide resistance genes (P450s). Linear regression analysis was used to test for a functional relationship between the resistance level of each insecticide (imidacloprid, thiamethoxam, and acetamiprid) and the mean normalized expression value of each P450s gene in different whitefly populations: **(A)** CYP6CX5, **(B)** CYP6CM1, **(C)** CYP6DZ4, **(D)** CYP6CX3, **(E)** CYP6CX1, **(F)** CYP4C64, **(G)** CYP6DZ7, and **(H)** CYP6DW2. Circles in each graph represent the gene expression values (marked on the left *Y*-axis), while squares depict the resistance ratio of each insecticide (labeled on the right *Y*-axis).

The correlation coefficient between RR values of the whitefly field population and the relative expression of detoxifying genes (P450s) is presented in Figures 7A–H. The expression level of CYP6CM1 significantly correlated with the RR of imidacloprid and thiamethoxam. The correlation coefficients were 0.94 (*p* = 0.012) and 0.95 (*p* = < 0.001), respectively (Figure 7B). The expression level of both CYP6CX1 and CYP6DW2 genes was positively correlated with the RR of acetamiprid (*R*^2^ = 0.72, *p* = 0.030; *R*^2^ = 0.91, *p* = 0.003) (Figures 7E,H).

Taking together, the RR of all the tested neonicotinoids exhibited a positive correlation with the transcript levels of CYP4C64 and CYP6DZ7 genes (Figures 7F,G). The correlation coefficients were 0.72 (*p* = 0.030) and 0.80 (*p* = 0.015) for imidacloprid, 0.69 (*p* =0.030) and 0.77 (*p* = 0.020) for thiamethoxam, and 0.75 (*p* = 0.020) and 0.79 (*p* = 0.010) for acetamiprid, respectively.

## Discussion

“Species concept” has been one of the most controversial concepts in biology consistently. This statement holds true for *B. tabaci*, as it is recognized as a complex of cryptic species that are morphologically indistinguishable from one another but genetically diverse (Firdaus et al., 2013; Hadjistylli et al., 2016). The Indian region exhibits an enormous diversity of *B. tabaci* with the presence of 10 cryptic species among the 46 species recorded so far (Rehman et al., 2021). The vast array of genetic groups not only specialize in host plant preference, but also exhibit a diverse dispersal pattern and differential response to pesticides (Pashley et al., 1992; Adamczyk et al., 1997; Barman et al., 2022). In this regard, precise assessment of the genetic identity and insecticide resistance properties is imperative for the development of control strategies.

An extensive survey of whitefly across different regions of Bengal province in India revealed that the pair-wise genetic variation reached up to 20.80%, and the species complex was further diversified into four cryptic species, namely, Asia I, Asia II 5, Asia II 7, and China 3. Of all the reported four genetic groups, Asia I was found to be the major group in the collected whitefly samples. This group was previously reported to be prevalent across Asian countries, including Pakistan, Bangladesh, Malaysia, Singapore, Indonesia, and Cambodia (Dinsdale et al., 2010; Götz and Winter, 2016; Kanakala and Ghanim, 2019). The Asia II cryptic whitefly species is a genetically diverse group consisting of 13 cryptic species, namely, Asia II (1–13) (Kanakala and Ghanim, 2019). Among them, the genetic groups of only Asia II 5 and Asia II 7 were detected in the collected whitefly samples spanning across the Bengal province. Previous reports suggest that Asia II 5 was majorly prevalent in the South Asian countries like Bangladesh, Pakistan, and Nepal (Islam et al., 2018; Khatun et al., 2018; Acharya et al., 2020). In the survey conducted, Asia II 5 was found to be majorly distributed along the southern part of Bengal and associated with a single host crop, that is, eggplant. From a geographical point of view, the close proximity of Bangladesh and Bengal provinces of India might have paved a way for the rapid dispersion of this particular genetic group in India. On the other hand, Asia II 7 was specifically identified in one region, Bagmundi, Purulia, indicating that this cryptic species did not undergo rampant dispersion. The distribution of China 3 was also restricted to only the Birbhum region of Bengal, aligning with the reports of Ellango et al. (2015). Despite the extensive survey, this genetic group did not extend its distribution until now. Keeping in mind the rampant distribution of different cryptic species along with the prevalence of suitable hosts, *B. tabaci* has a high chance of developing adaptive advantage in different regions of the country.

Management of whiteflies is a grave task for farmers and scientists all around the globe, with chemical pesticides being the primary strategy in diverse agricultural systems. After gradual replacement with different groups of insecticides, the neonicotinoids, particularly imidacloprid, are extensively used against *B. tabaci* (Nauen and Denholm, 2005; Barman et al., 2021). Existing field problems, such as poor selection of insecticides and sub-standard application techniques, result in control failures of insecticides against this pest (Peshin and Zhang, 2014; Wang et al., 2020). Several global studies have highlighted the development of resistance in different whitefly populations to different groups of insecticides (Zhao et al., 2020). However, the occurrence of different cryptic forms makes the task further complex due to differential responses to insecticides. Furthermore, the increasing level of resistance observed in the whitefly population and the rapid evolution of insecticide resistance genes are also a matter of concern. Nonetheless, literature focusing on the susceptibility/resistance status of the whitefly population to insecticides in India is also limited. In this regard, field populations of whitefly surveyed from different geographical locations were tested for their susceptibilities to three commonly used neonicotinoids in these regions. The results revealed that among the three studied insecticides (thiamethoxam, imidacloprid, and acetamiprid), the high susceptibility of the population was recorded toward thiamethoxam. Variation in susceptibility status across different regions can be attributed to the prevalence of different genetic groups. The study data show that the whitefly population from Purulia and Hooghly, comprising Asia II 7 and Asia II 5 cryptic species, recorded higher resistance toward these novel compounds. Several global studies have documented resistance in *B. tabaci* MED and MEAM 1 genetic groups to different groups of insecticides across the continent (Whalon et al., 2013). However, there is limited literature available on the insecticide status of indigenous *B. tabaci* genetic groups in Asia. This study clearly provides the insecticide resistance/susceptibility status of Asian genetic groups like Asia I, Asia II 5, and Asia II 7 against the selected neonicotinoid compounds. As insecticide resistance is regarded by some workers as a major driving force for the selection and establishment of specific *B. tabaci* genetic groups in a region (Horowitz et al., 2005; Barman et al., 2021), there is a need for regular monitoring of insecticide resistance status in diverse *B. tabaci* genetic groups across India. Results from the present survey indicate the dominance of Asia I groups in most of the regions, with Asia II 5 ranking second on the basis of abundance. What is evident in the coming times is that if Asia II 5 or Asia II 7 becomes dominant in these regions, management using neonicotinoids would eventually become difficult. Therefore, further studies were carried out for unraveling the basis of insecticide resistance in different *B. tabaci* field populations, which is necessary for the future management of this pest.

Furthermore, endosymbionts also have an important role to play in protecting their hosts from different environmental stressors, including insecticides (Bhatt et al., 2021). This symbiont-mediated insecticide resistance or susceptibility is highly variable and depends on the species of symbiont and the chemical compound (Liu and Guo, 2019). Hence, the work further focussed on bacterial endosymbiont communities which infect different *B. tabaci* populations across a wide geographical range to gain a detailed understanding of the endosymbiont diversity and their association with insecticide resistance. The profiles of endosymbionts within the different whitefly genetic groups were highly variable. A significant positive association of *Wolbachia* titer was observed with the resistance level of the three test insecticides (imidacloprid, thiamethoxam, and acetamiprid) in different whitefly field populations. Earlier reports opined that the presence of *Wolbachia* increased the resistance of whiteflies to neonicotinoids, such as acetamiprid (Ghanim and Kontsedalov, 2009). On the other hand, *Arsenophonus* also correlated well with the resistance levels of imidacloprid and thiamethoxam. Although the co-existence of *Arsenophonus* with *Rickettsia* symbiont was reported to confer resistance to acetamiprid (Kontsedalov et al., 2008), no such significant correlation between *Rickettsia* titer and insecticide resistance level in whitefly was noted. Such a difference in symbiont performance can be attributed to symbiont species, host genotype, and co-infection with other symbionts (Ghanim and Kontsedalov, 2009; Hoffmann et al., 2015). The network analysis used to compare the genetic variation of secondary symbionts (study samples) with other Asian countries revealed considerable overlap between the haplotypes (Indonesia, Korea, China, Bangladesh, and Pakistan), sometimes with a little variation. These results are clearly suggestive of the fact that the symbionts of whitefly are effectively isolated from other locations and might be in the process of genetic divergence. In contrast, there appear to be significant interactions between the whitefly populations and other distant countries despite the stretches of kilometers between them. The mixing of whitefly populations seems unavoidable due to the migration of nymphs that can occur up to 7 km (Byrne et al., 1996). Hence, this is a source of concern, as it raises the possibility of resistance build up in whiteflies of other countries as well, provided the symbiont dynamics remain the same.

Overexpression and modification of gene encoding members of cytochrome P450, carboxylesterases, and glutathione S-transferases (GSTs) are associated with resistance to different groups of insecticides (Li et al., 2007). In the current study, expression of both *CYP6DZ7* and *CYP4C64* showed a good correlation with the resistance level of imidacloprid, thiamethoxam, and acetamiprid in different whitefly field populations. According to previous reports, expression of *CYP4C64* positively correlated with imidacloprid resistance in whitefly; however, no such correlation was recorded in *CYP6DZ7* (Yang et al., 2013). Possibly, the expression of *CYP6DZ7* imparts resistance to imidacloprid in some whitefly field populations but not in others. As reported by Zhuang et al. (2011), *CYP6CX1* is involved in imidacloprid resistance in both laboratory and field populations of whitefly. Present results revealed a direct correlation of *CYP6CX1* with acetamiprid resistance. Based on the current and previous studies, it can be presumed that *CYP6CX1* is an important gene in conferring resistance to whitefly against several neonicotinoid compounds. Moreover, similar to earlier reports, *CYP6CM1* expression also correlated absolutely well with the resistance level of imidacloprid and thiamethoxam. Therefore, multiple symbiotic interactions with varying gene expression patterns might govern the differential susceptibility of the whitefly population against different test insecticides.

Assembling together all these pieces of information, this research article provides an in-depth notion of the current scenario of different whitefly cryptic species and their susceptibility status toward the selected neonicotinoids. The study further dwells on the infection patterns and role of endosymbionts in imparting insecticide resistance. Studies of symbiont-mediated resistance could provide an interesting prospect to determine biological phenomenona with implications that may extend beyond entomological research and further contribute to a greater understanding of bioremediation, human–microbiome interactions, pharmacology, etc. The use of neonicotinoid group of insecticides is employed as a major strategy for controlling insects like whiteflies; however, the deep-rooted negative environmental effects of neonicotinoids call for serious attention to some alternative management techniques for this pest. With further development, bioaugmentation of detoxifying bacteria could possibly serve to reduce pesticide residuals in the environment, henceforth improving the health of the ecosystem while eliminating the deleterious effects of these pesticides.

Furthermore, an understanding of different P450 genes and their positive correlation with insecticide resistance in different whitefly populations have also been depicted. Nonetheless, the heterogeneity observed in the collected whitefly population indicates toward the differential response it might show toward the management approaches, which is indeed a matter of concern for scientists and farming communities. A few vital questions that arise on our way stem from the basic evolution of symbiont-mediated insecticide resistance and the precise molecular interactions occurring between symbiont and host genes. Possibly through proper clarification of these vital issues, symbiont-targeted pest management will prove to be an effective control component for the future agricultural community. However, detailed basic work needs to be carried out before the large-scale application of symbiont-targeted pest management.

## Data Availability Statement

The original contributions presented in the study are included in the article/Supplementary Material, further inquiries can be directed to the corresponding author/s.

## Author Contributions

Conceptualization: MB and SS. Methodology: MB and HT. Original draft preparation and writing—reviewing and editing the manuscript: SS and GU. Validation: MB and GU. Resources: AS and JT. Investigation: JT and SC. All authors have read and agreed to the published version of the manuscript.

## Conflict of Interest

The authors declare that the research was conducted in the absence of any commercial or financial relationships that could be construed as a potential conflict of interest.

## Publisher's Note

All claims expressed in this article are solely those of the authors and do not necessarily represent those of their affiliated organizations, or those of the publisher, the editors and the reviewers. Any product that may be evaluated in this article, or claim that may be made by its manufacturer, is not guaranteed or endorsed by the publisher.
