# Supplementary material for: Unraveling the Basis of Neonicotinoid Resistance in Whitefly Species Complex: Role of Endosymbiotic Bacteria and Insecticide Resistance Genes
Source: Front Microbiol. 2022 Jun 23;13:901793. doi: 10.3389/fmicb.2022.901793 (PMC9260502; doi:10.3389/fmicb.2022.901793)
Supplement: Supplementary file 1 [file Data_Sheet_1.docx]

**Unravelling the basis of neonicotinoid resistance in whitefly species complex: role of endosymbiotic bacteria and insecticide resistance genes**

Mritunjoy Barman^1†^, Snigdha Samanta^1†^, Gouranga Upadhyaya^2*^, Himanshu Thakur^3^, Swati Chakraborty^4^, Arunava Samanta^1^, Jayanta Tarafdar ^4*^

**^1^** Department of Agricultural Entomology, Bidhan Chandra Krishi Viswavidyalaya, West Bengal, India.

**^2^** Department of Biological Sciences, Indian Institute of Science Education and Research Kolkata, West Bengal 741246.

**^3^** Department of Entomology, C.S.K. Himachal Pradesh Krishi Vishvavidyalaya, Palampur, Himachal Pradesh, India

**^4^** Department of Plant Pathology, Bidhan Chandra Krishi Viswavidyalaya, Nadia, West Bengal, India.

*Corresponding author

Email: jayanta94bckv@gmail.com, gour.cubot@gmail.com; Telephone: +919830342320, +919547414909

^†^ These authors have contributed equally to this work and shares first authorship

**Table S1:** Collecting information of whitefly (*B. tabaci*) from different location of Bengal provinces, India.

| **SAMPLE ID** | **Collection sites** | **Location coordinates** | **Host plant** | **Collection Date** | **Cryptic species** | **Secondary symbionts** | | |
| --- | --- | --- | --- | --- | --- | --- | --- | --- |
|  |  |  |  |  |  | **A** | **W** | **R** |
| **1. HAW-I** | Bally, Hawrah | 22.6497° N, 88.3386° E | Bhendi (*Abelmoschus esculentus)* | Feb, 2021 | **Asia-I** | A1 | W1 | _ |
| **2.HOG-II** | Pandua, Hooghly | 23.0781° N, 88.2789° E | Pointed gourd (*Trichosanthes dioica* Roxb.) | Feb, 2021 | **Asia-I** | A1 | W1 | R3 |
| **3.MLD-1** | English bazar, Malda | 25.0108° N, 88.1411° E | Cucumber (*Cucumis sativus*) | Mar, 2021 | **Asia-I** | A2 | W1 | R3 |
| **4.ALP- II** | Birparda, Aliporeduar | 26.7058° N, 89.1373° E | Brinjal (*Solanum melongena* L.) | Mar, 2021 | **Asia-I** | A1 | _ | R3 |
| **5.KP-I** | Rongo Forest, Kalingpong | 27.0435°N, 88.8335°E | Sida (*Sida acuta*) | Mar, 2021 | **Asia-I** | A2 | W1 | R3 |
| **6.KP-II** | Gairibas, Kalinpong | 26.6299°N, 88.38363°E | Tomato (*Solanum lycopersicum* ) | Mar, 2021 | **Asia-I** | A2 | W1 | R3 |
| **7.PHO-I** | Chandannagar, Hooghly | 22.8671° N, 88.3674° E | Brinjal (*Solanum melongena* L.) | Mar, 2020 | **Asis-II-5** | A2 | W1 | R3 |
| **8.JS4P** | Jaynagar, South 24 Parganas | 22.1742° N, 88.4234° E | Brinjal (*Solanum melongena* L.) | Feb 2021 | **Asis-II-5** | A1 | _ | _ |
| **9.BR-I** | Nalhati, Birbhum | 24.2921° N, 87.8378° E | Cucumber (*Cucumis sativus*) | Dec, 2020 | **China 3** | _ | W1 | R3 |
| **10.JLG-II** | Mynaguri, Jalpaiguri | 26.5738° N, 88.8215° E | Brinjal (*Solanum melongena* L.) | Mar, 2020 | **Asis-II-5** | A2 | _ | _ |
| **11.HN24** | Habra, N 24 Parganas | 22.8378° N, 88.65375° E | Brinjal (*Solanum melongena* L.) | Feb, 2021 | **Asia-I** | _ | _ | R3 |
| **12.BN24** | Basirhat, N 24 Parganas | 26.6472° N , 88.86718 °E | Brinjal (*Solanum melongena* L.) | Feb, 2021 | **Asis-II-5** | _ | W1 | _ |
| **13.PUR-I** | Baghmundi, Purulia | 23.1931° N, 86.0504° E | Brinjal (*Solanum melongena* L.) | Dec, 2020 | **Asia-II-7** | A1 | W1 | R3 |
| **14.SDP-II** | Gangarampur, South Dinajpur | 25.4009° N, 88.5324° E | Wild Brinjal (*Solanum torvum)* | Mar, 2020 | **Asia-I** | _ | W1 | _ |
| **15.MD-I** | Mayna, Malda | 25.2931° N, 88.1609° E | Tomato (*Solanum lycopersicum* ) | Dec, 2020 | **Asia-I** | A1 | _ | _ |
| **16.MID-2** | Kharagpur, Midnapore | 25.0778° N, 87.9004° E | Sida (*Sida acuta*) | Mar, 2021 | **Asia-I** | A1 | W1 | R3 |
| **17.MD-III** | Kaliachak, Malda | 24.8602° N, 88.0192° E | Tomato (*Solanum lycopersicum* ) | Mar, 2021 | **Asia-I** | A1 | _ | _ |
| **18.GMID-I** | Ghatal, Midnapur | 22.6637° ‎N, ‎87.7468°E | Brinjal (*Solanum melongena* L.) | Mar, 2021 | **Asis-II-5** | A1 | _ | _ |
| **19.NDP-I** | Kalyani, Nadia | 22.9747° N, 88.4337° E | Sida (*Sida acuta*) | Mar,2020 | **Asia-I** | A1 | W1 | R3 |
| **20.NDP-II** | Modanpur, Nadia | 23.0089° N, 88.4912° E | Wild Brinjal (*Solanum torvum)* | Mar, 2020 | **Asia-I** | A1 | _ | R3 |
| **21.SDP-I** | Kumarganj, South Dinajpur | 25.4249° N, 88.7289° E | Brinjal (*Solanum melongena* L.) | Mar, 2020 | **Asia-I** | A1 | _ | _ |
| **22.COB-I** | Tufanganj, Coochbehar | 26.3035° N, 89.5946° E | Brinjal (*Solanum melongena* L.) | Mar, 2020 | **Asia-I** | A2 | W1 | R3 |
| **23.COB-II** | Pundibari, Coochbehar | 26.29657° N, 89.19987° E | Cucumber (*Cucumis sativus*) | Mar, 2020 | **Asia-I** | A2 | _ | R3 |
| **24.JLG-I** | Dhupguri, jalpaiguri | 26.5821° N, 89.0051° E | Brinjal (*Solanum melongena* L.) | March, 2020 | **Asia-I** | _ | _ | R3 |
| **25.BR-II** | Bolpur, Birbhum | 23.6712° N, 87.6919° E | Pointed gourd (*Trichosanthes dioica* Roxb.) | Dec, 2020 | **China 3** | _ | W1 | R3 |
| **26.WB-I** | Rajnagar, Birbhum | 23.9451° N, 87.3140° E | Brinjal (*Solanum melongena* L.) | Jan, 2021 | **China 3** | _ | _ | R3 |

**Table S2:** List of primers used in the study.

| **Organism** | **Primer Name** | **Primer Sequences (5′→3′)** | **Annealing Temperature (°C)** |
| --- | --- | --- | --- |
| **Diagnostic PCR** | | | |
| ***Portiera*** | Por-F  Por-R | TGCAAGTCGCGGCATCAT  CCGCCTTCTGCGTTGGCAACT | 54 |
| ***Arsenophonus*** | Arse-F  Arse-R | CGTTTGATGAATTCATAGTCAAA  GGTCCTCCAGTTAGTGTTACCCAAC | 54 |
| ***Rickettsia*** | Ric-F Ric-R | GCTCAGAACGAACGCTGG  GAAGGAAAGCATCTCTGC | 56 |
| ***Wolbachia*** | Wol-F Wol-R | CGGGGGAAAATTTATTGCT  AGCTGTAATACAGAAAGGAAA | 56 |
| ***B. tabaci*** | C1-J-2195  L2-N-3014 | TTGATTTTTTGGTCATCCAGAAGT  TCCAATGCACTAATCTGCCATATTA | 52 |
| **qRT PCR** | | | |
| ***Portiera*** | Port73-F  Port266-R | TAGTCCACGCTGTAAACG  AGGCACCCTTCCATCT | 60 |
| ***Rickettsia*** | glt375-F  glt574-R | AAAGGTTGCTCATCATGCGTT  GCCATAGGATGCGAAGAGCT | 60 |
| ***Wolbachia*** | Wsp-F  Wsp-R | TGGTCCAATAAGTGATGAAGAAAC  AAAAATTAAACGCTACTCCA | 60 |
| ***Arsenophonus*** | 23S-F  23S-R | CGTTTGATGAATTCATAGTCAAA  GGTCCTCCAGTTAGTGTTACCCAAC | 60 |
| **CYP6CX5** | CX5  CX5 | GACTTTCCAGCTGCTCAACCC  GTTCCCGCTGAGCTTGTCCA | 60 |
| **CYP6CM1** | CM1  CM1 | CACTCTTTTGGATTTACTGC  GTGAAGCTGCCTCTTTAATG | 60 |
| **CYP6DZ4** | DZ4-F  DZ4-R | AGGATGGTCACGCTAAGGATG  AGTGTGGTGGCAGTTGTCTC | 60 |
| **CYP6CX3** | CX3-F  CX3-R | CGCATTCTTCCAGTTCCTCGAGA  GGCCATAGCATCCTTCGTGACC | 60 |
| **CYP6CX1** | CX1-F  CX1-R | GTGCCCTACATCTCGCCTATC  CATTTCTTTCGTCGTCTCCAAC | 60 |
| **CYP4C64** | C64-F  C64-R | TCGGATTACGTCAGAGCTATTTAC  GTGGAGCACGCTTAGACA | 60 |
| **CYP6DZ7** | DZ7-F  DZ7-R | CTGTCTACGGTCTCCATC  CCGAAAGGCAGATACGAT | 60 |
| **CYP6DW2** | DW2-F  DW2-R | CAGGCGAGCAAACCGTATC  GGTGAAGTGCGAGAAGTCC | 60 |
| **Actin** | Actin-F  Actin-R | ACCGCAAGATTCCATACCC  CGCTGCCTCCACCTCATT | 60 |

**Table S3:** List of *B. tabaci* field populations used in bioassay.

| **Populations** | **Geographic origin**  **(Agro-climatic zone - States)** | **GPS coordinates** | **Host plant** | **Identification**  **(Genetic group)** | **Endosymbionts** | | | |
| --- | --- | --- | --- | --- | --- | --- | --- | --- |
|  |  |  |  |  | ***C. portiera*** | ***Arsenophonus*** | ***Wolbachia*** | ***Rickettsia*** |
| 1. Midnapore | Coastal and Saline Zone | 22.6637° ‎N, ‎87.7468°E | Brinjal | **Asia I** | OK036339 | OK042290 | MZ871371 | OK036575 |
| 2.Hooghly | Old Alluvial Zone | 22.8671° N, 88.3674° E | Brinjal | **Asia II 5** | OK036336 | MZ853774 | MZ871369 | OK036572 |
| 3.Kalimpong | Hill Zone | 26.6299°N, 88.38363°E | Tomato | **Asia I** | OK036337 | MZ853775 | MZ871368 | OK036573 |
| 4.Malda | Old Alluvial Zone | 25.0108° N, 88.1411° E | Cucumber | **Asia I** | OK036338 | MZ853776 | MZ871370 | OK036574 |
| 5.Purulia | Red and Laterite Zone | 23.1931° N, 86.0504° E | Brinjal | **Asia II 7** | OK036341 | MZ853777 | MZ871372 | OK036577 |
| 6.Nadia | New Alluvial Zone | 22.9747° N, 88.4337° E | Sida | **Asia I** | OK036340 | MZ853778 | MT032316 | OK036576 |

**Table S4:** Pairwise comparisons of the intraspecific and interspecific variation in cytochrome oxidase subunit 1 (*COI*) nucleotide sequences among the four identified cryptic *B.tabaci* species

| Cryptic species | Asia I | Asia II 5 | China 3 | Asia II 7 |
| --- | --- | --- | --- | --- |
| Asia I | 0.24-2.66% |  |  |  |
| Asia II 5 | 17.47-20.80% | 0.11-1.44% |  |  |
| China 3 | 14.63-17.09% | 16.05-17.28% | 0.11-0.23% |  |
| Asia II 7 | 16.56-17.39% | 12.28-13.11% | 16.95-17.30% | - |

**Table S5:** Comparison of the genetic structure of cytochrome oxidase subunit I *(COI)* sequences among the four identified cryptic *B.tabaci* species.

| **Analysis** | **Cryptic species** | | | |
| --- | --- | --- | --- | --- |
|  | **Asia I** | **Asia II 5** | **China 3** | **Asia II 7** |
| **Sample size** | 17 | 5 | 3 | 1 |
| **Number of Haplotypes** | 12 | 5 | 2 | 1 |
| **Haplotype diversity (Hd)** | 0.956 | 1.00 | 0.500 | - |
| **Nucleotide diversity (π)** | 0.014 | 0.008 | 0.001 | - |
| **Number of polymorphic (segregating) sites (S)** | 43 | 15 | 2 | - |
| **Fu and Li’s D*** | 0.706 (P> 0.10) | -0.203 * | -0.604* | - |
| **Fu and Li’s F*** | 0.464 (P> 0.10) | -0.216* | -0.604* | - |
| **Fu’s F** | -0.531 | -0.752 | 1.099 | - |
| **Tajima’s D** | -0.354 (P> 0.10) | -0.203* | -0.709* | - |

**Table S6:** Genetic variance of *B.tabaci* cryptic species.

| **Source of variation** | **d.f** | **Sum of squares** | **Variance components** | **Percentage variation** | **Fixation indices (F-statistics)** |
| --- | --- | --- | --- | --- | --- |
| **Among populations** | 3 | 1571.03 | 56.89 | 91.83 | F_IS_ : 1.00 |
| **Within populations** | 22 | 222.73 | 5.06 | 8.16 | F_ST_ : 0.91 |
| **Total** | 25 | 1793.76 | 61.96 | _ | _ |

**Table S7.** Pairwise comparison of genetic distance (Fst) between four cryptic species.

| Cryptic species | Asia I | Asia II 5 | China 3 | Asia II 7 |
| --- | --- | --- | --- | --- |
| Asia I | **-** |  |  |  |
| Asia II 5 | **0.92** | **-** |  |  |
| China 3 | **0.91** | **0.96** | **-** |  |
| Asia II 7 | **0.90** | **0.93** | **0.99** | **-** |

**Table S8**: Infection rates of secondary symbionts in four cryptic species of *B. tabaci* from different locations.

| **Secondary endosymbionts** | **Infection rates [ % (individual numbers)] of each cryptic species** | | | | | |
| --- | --- | --- | --- | --- | --- | --- |
|  | **Sub-groups** | **Asia I** | **Asia II 5** | **China 3** | **Asia II 7** | **Overall** |
| ***Arsenophonus*** | A1 | 52.94 % (9) | 20% (1) | - | 100 % (1) | 42.30% (11) |
| _ | A2 | 35.29 % (6) | 40% (2) | - | - | 30.76 % (8) |
| _ | A1+A2 | 88.23% (15) | 60% (3) | - | 100 % (1) | 73.07% (19) |
| ***Wolbachia*** | W1 | 52.94 % (9) | 40% (2) | 66.67% (2) | 100 % (1) | 53.84% (14) |
| ***Rickettsia*** | R3 | 70.58 % (12) | 20% (1) | 100% (3) | 100 % (1) | 65.38% (17) |
| **Total number of whitefly individuals** | _ | 17 | 5 | 3 | 1 | 26 |

**Table S9:** Multiple infection pattern of secondary endosymbionts in four cryptic species of *B.tabaci*.

| **Combinations of endosymbionts** | **Infection rates [ % (individual numbers)] of each cryptic species** | | | |
| --- | --- | --- | --- | --- |
|  | **Asia I** | **Asia II 5** | **China 3** | **Asia II 7** |
| **A1W1** | 5.88%(1) | - | - | - |
| **A1W1R3** | 17.64%(3) | - | - | 5.88%(1) |
| **A2W1R3** | 23.52%(4) | 5.88%(1) | - | - |
| **A1R3** | 11.76%(2) | - | - | - |
| **A1** | 17.64%(3) | 11.76%(2) | - | - |
| **W1R3** | - | - | 66.66 %(2) | - |
| **A2** | - | 5.88%(1) | - | - |
| **R3** | 11.76%(2) | - | 5.88%(1) | - |
| **W1** | 5.88%(1) | 5.88%(1) | - | - |
| **A2R3** | 5.88%(1) | - | - | - |
| **Total no. of whiteflies** | 17 | 5 | 3 | 1 |

**Table S10:** Neutrality tests for 23S and 16S rDNA sequences of *Arsenophonus* sp., *Wolbachia* sp., and *Rickettsia* sp.

| **Analysis** | ***Arsenophonus*** | | ***Wolbachia*** | | ***Rickettsia*** | |
| --- | --- | --- | --- | --- | --- | --- |
|  | **Sequences used in current study** | **Sequences outside India** | **Sequences used in current study** | **Sequences outside India** | **Sequences used in current study** | **Sequences outside India** |
| **Sample size** | 19 | 45 | 14 | 18 | 17 | 21 |
| **Number of Haplotypes** | 6 | 19 | 5 | 11 | 6 | 17 |
| **Haplotype diversity (Hd)** | 0.857 | 0.917 | 0.803 | 0.856 | 0.879 | 0.967 |
| **Nucleotide diversity (π)** | 0.028 | 0.031 | 0.006 | 0.132 | 0.003 | 0.028 |
| **Number of polymorphic (segregating) sites (S)** | 47 | 93 | 13 | 151 | 9 | 110 |
| **Fu and Li’s D*** | 0.351 (P> 0.10) | -1.720 (P> 0.10) | 0.536 (P>0.10) | 0.427 (P>0.10) | 0.108 (P> 0.10) | 0.588 (P> 0.10) |
| **Fu and Li’s F*** | 0.298 (P> 0.10) | -1.9(0.10 > P> 0.05) | 0.292 (P>0.10) | 0.135 (P>0.10) | 0.093 (P> 0.10) | 0.214 (P>0.10) |
| **Fu’s F** | 6.767 | 0.631 | 1.360 | 5.748 | -0.307 | -0.755 |
| **Tajima’s D** | 0.295 (P> 0.10) | -1.425 (P> 0.10) | -0.538 (P>0.10) | -0.628 (P>0.10) | 0.006 (P> 0.10) | -0.735 (P> 0.10) |

**Table S11:** Susceptibility of whitefly, *B. tabaci* field populations to three neonicotinoid insecticides.

| **Insecticide** | **Location** | **LC_50_ (mg/L)** | **95% C.L.** | **RR at LC_50_** | **Slope±SE** | **χ^2^**  **(*p*)** | **Resistance category** |
| --- | --- | --- | --- | --- | --- | --- | --- |
| **Thiamethoxam** | Laboratory | 5.36 | 1.11-14.00 | 1.00 | 0.53±0.11 | 1.20  (0.75) | Susceptible |
|  | Midnapore | 9.46 | 1.88-26.84 | 1.76 | 0.47±0.11 | 0.95  (0.81) | Low Resistance |
|  | Hooghly | 34.61 | 10.01-124.40 | 6.46 | 0.44±0.11 | 1.37  (0.71) | Moderate  Resistance |
|  | Kalimpong | 13.35 | 2.82-39.79 | 2.49 | 0.44±0.11 | 1.06  (0.79) | Low Resistance |
|  | Malda | 17.60 | 4.08-54.69 | 3.28 | 0.44±0.11 | 1.50  (0.68) | Low Resistance |
|  | Purulia | 52.14 | 15.13-248.03 | 9.73 | 0.41±0.11 | 1.48  (0.69) | Moderate  Resistance |
| **Imidacloprid** | Laboratory | 12.87 | 2.62-33.51 | 1.00 | 0.51±0.12 | 5.20  (0.16) | Susceptible |
|  | Midnapore | 26.23 | 6.21-74.28 | 2.04 | 0.47±0.12 | 3.84  (0.28) | Low Resistance |
|  | Hooghly | 77.79 | 25.11-315.70 | 6.04 | 0.45±0.12 | 2.49  (0.48) | Moderate  Resistance |
|  | Kalimpong | 29.99 | 8.54-79.35 | 2.33 | 0.51±0.12 | 2.73  (0.44) | Low Resistance |
|  | Malda | 44.53 | 11.48-153.83 | 3.46 | 0.44±0.12 | 2.08  (0.56) | Low Resistance |
|  | Purulia | 112.09 | 38.66-548.81 | 8.71 | 0.46±0.13 | 1.83  (0.61) | Moderate  Resistance |
| **Acetamiprid** | Laboratory | 13.49 | 3.91-27.57 | 1.00 | 0.68±0.17 | 3.58  (0.31) | Susceptible |
|  | Midnapore | 41.59 | 19.01-98.32 | 3.08 | 0.68±0.17 | 3.75  (0.29) | Low Resistance |
|  | Hooghly | 60.04 | 29.48-165.26 | 4.45 | 0.69±0.18 | 3.98  (0.26) | Low Resistance |
|  | Kalimpong | 34.30 | 13.53-84.08 | 2.54 | 0.62±0.17 | 2.99  (0.39) | Low Resistance |
|  | Malda | 24.37 | 8.10-55.42 | 1.81 | 0.61±0.17 | 3.18  (0.36) | Low Resistance |
|  | Purulia | 52.23 | 22.76-150.70 | 3.80 | 0.62±0.17 | 3.12  (0.37) | Low Resistance |
